# *Sinodraconarius* gen. n., a new genus of Coelotinae spiders from Southwest China (Araneae, Agelenidae)

**DOI:** 10.3897/zookeys.770.22470

**Published:** 2018-07-04

**Authors:** Bing Li, Zhe Zhao, Chuntian Zhang, Shuqiang Li

**Affiliations:** 1 College of Life Science, Shenyang Normal University, Shenyang, Liaoning 110034, China; 2 Institute of Zoology, Chinese Academy of Sciences, Beijing 100101, China

**Keywords:** Asia, taxonomy, new combination, new species

## Abstract

A new genus of the subfamily Coelotinae F.O. Pickard-Cambridge, 1893, *Sinodraconarius* **gen. n.**, with four new species, *S. cawarongensis* **sp. n.** (♂♀), *S. muruoensis* **sp. n.** (♂♀), *S. sangjiuensis* **sp. n.** (♂♀, type species), *S. yui* **sp. n.** (♂♀) and *S. patellabifidus* (Wang, 2003) **comb. n.**, ex. *Draconarius* Ovtchinnikov, 1999 is described. The genus is restricted to Southwest China. *Sinodraconarius* **gen. n.** is most similar to *Draconarius* but can be distinguished by the shape of the copulatory organs. The DNA barcodes of all species were documented for future use.

## Introduction

The spider subfamily Coelotinae (Araneae, Agelenidae) comprises 694 valid species belonging to 27 genera worldwide (World Spider Catalog 2018), of which 88% of the species are restricted to Asia, 8% to Europe and 4% to North America. So far, 339 coelotine species in 21 genera are known from China, including three genera erected in recent years: *Flexicoelotes* Chen, Li & Zhao, 2015, *Papiliocoelotes* Zhao & Li, 2016 and *Sinocoelotes* Zhao & Li, 2016.

*Draconarius* Ovtchinnikov, 1999, with 246 named species, is the largest genus of Coelotinae. Recent molecular studies suggested that *Draconarius* is polyphyletic and requires taxonomic rearrangements (Zhao and Li 2017). Here, we described a new genus, *Sinodraconarius* gen. n., with four new species.

## Material and methods

The specimens were examined with a LEICA M205C stereomicroscope. The photographs were captured with an Olympus C7070 wide zoom digital camera (7.1 megapixels) mounted on an Olympus SZX12 dissecting microscope and an Olympus BX51 compound microscope. Photos from multiple focal planes were combined using Helicon Focus (Version 3.00) photo stacking software. Epigynes and male palps were examined after dissection from the spiders’ bodies. Epigynes were cleared by boiling in a 10% potassium hydroxide (KOH) water solution before taking photos of the vulva.

All measurements were obtained using a LEICA M205C stereomicroscope and are in millimeters. Eye sizes were measured as the maximum diameter from either the dorsal or frontal views. Leg measurements are given as: total length (femur, patellatibia, metatarsus, tarsus). The male palps depicted are the left ones. The terminology used in the text and figures follows Wang (2002). Abbreviations:

Morphological characters:

**A** epigynal atrium;

**ALE** anterior lateral eye;

**AME** anterior median eye;

**AME–ALE** distance between AME and ALE;

**AME–AME** distance between AME and AME;

**AME–PME** distance between AME and PME;

**ALE–PLE** distance between ALE and PLE;

**C** conductor;

**CD** copulatory duct;

**CDA** conductor dorsal apophysis;

**CF** cymbial furrow;

**CO** copulatory opening;

**E** embolus;

**EB** embolic base;

**FD** fertilization duct;

**LTA** retro-lateral tibial apophysis;

**MA** median apophysis;

**PA** patellar apophysis;

**PLE** posterior lateral eye;

**PME** posterior median eye;

**PME–PLE** distance between PME and PLE;

**PME–PME ** distance between PME and PME;

**R** receptacle;

**RTA** retroventral tibial apophysis;

**ST** subtegulum;

**T** tegulum.

DNA barcodes were obtained for future use. A partial fragment of the mitochondrial cytochrome oxidase subunit I (COI) gene was amplified and sequenced for all species, using the following primers: Forward: LCO1490-oono (5’-CWACAAAYCATARRGATATTGG-3’) and Reverse: C1-N-2776 (5’-GGATAATCAGAATANCGNCGAGG-3’). For additional information on extraction, amplification and sequencing procedures, see Zhao and Li (2017). All sequences were analyzed using BLAST and are deposited in GenBank. The accession numbers are provided in Table 1.

**Table 1.** Voucher specimen information.

Species	GenBank accession number	Sequence length	Collection localities
*S. cawarongensis* sp. n.	KY778914	1194bp	Zhowagoin Township, Zayü, Tibet, China
*S. muruoensis* sp. n.	KY778913	1194bp	Zhowagoin Township, Zayü, Tibet, China
*S. patellabifidus*	KY778910	1194bp	Liuku Township, Lushui, Yunnan, China
*S. sangjiuensis* sp. n.	KY778915	1194bp	Zhowagoin Township, Zayü, Tibet, China
*S. yui* sp. n.	KY778908	1194bp	Segula Mountain, Nyingchi, Tibet, China

All of the specimens (including molecular vouchers) are deposited in the Institute of Zoology, Chinese Academy of Sciences (IZCAS), Beijing, China.

## Taxonomy

### Family Agelenidae C.L. Koch, 1837

#### Subfamily Coelotinae F.O. Pickard-Cambridge, 1893

##### Genus Sinodraconarius

Taxon classificationAnimaliaAraneaeAgelenidae

Z. Zhao & S. Li gen. n.

http://zoobank.org/8FD70171-B9AF-49D7-967B-74B3AEB9945E

###### Type species.

*Sinodraconarius sangjiuensis* Zhao & Li, sp. n.

###### Etymology.

The generic name is derived from its similarity to *Draconarius* and the Latin adjective *Sino*- for Chinese referring to the main distribution region of the genus. The gender is masculine.

###### Diagnosis.

The males of *Sinodraconarius* gen. n. are similar to those of *Draconarius* by having a patellar apophysis, two tibial apophyses (RTA and LTA) and a long median apophysis, but can be distinguished by the short cymbial furrow, less than 1/2 length of cymbium *vs.* long and generally more than 1/2 length of the cymbium in *Draconarius*; patellar apophysis bifurcate *vs.* not bifurcate in *Draconarius*. The females of *Sinodraconarius* gen. n. are similar to those of *Draconarius* by having a small epigynal atrium, with epigynal hoods located laterally, and the copulatory openings located centrally on the epigyne plate, but can be distinguished by lacking epigynal teeth; receptacles simple.

###### Description.

Small to very large sized, with a total length of 6.90–17.60; body brownish to brown, with black setae. Carapace nearly pear-shaped, with longitudinal fovea and radial grooves; sternum brownish, heart-shaped. Abdomen nearly oval, grey to dark grey, with 4–5 grey chevron-like markings. Chelicerae with three promarginal and two retromarginal teeth. Leg formula (4 > 1 > 2 > 3). Male palp with one bifurcate patellar apophysis; two tibial apophyses (RTA and LTA), RTA extending beyond the tibia; cymbial furrow short, less than 1/2 length of cymbium; conductor short, with dorsal conductor apophysis; the apex of conductor with small basal lamella; embolus short; median apophysis long, finger-like; tegulum broad. Tibia strongly bent and dorsal part of tibia and patella bent almost to a right angle, ventral part of tibia at 45° angle. Epigyne: with septum; teeth lacking; atrium small, length of atrium two times longer than width, heart-shaped; epigynal hoods located laterally; copulatory openings located centrally on epigynal plate; copulatory ducts short, extending mesad of receptacles; receptacles broad, widely separated.

###### Comments.

In addition to morphological study, we analyzed the relationships of coelotine spiders using eight genes from 286 species in 19 genera (Zhao and Li 2017). The molecular topologies inferred by three different approaches all supported *Sinodraconarius* gen. n. as a monophyletic group that is closely related to *Draconarius*. For details, please see SD001, SD002, SD019, SD028 and ZZ300 (Southern *Coelotes* groups) in Figure 3 and supplementary figures S4–S6 of Zhao and Li (2017).

###### Distribution.

So far, the genus is known from Tibet and Yunnan, China (Fig. 11).

##### Sinodraconarius sangjiuensis

Taxon classificationAnimaliaAraneaeAgelenidae

Z. Zhao & S. Li sp. n.

http://zoobank.org/50956D57-E022-4FBB-8BE3-F73C258B3441

Figs 1 , 2 , 11

###### Type material.

**Holotype** ♂ (IZCAS): China: Tibet: Zayü: Zhowagoin Township, Sangjiu Village, Mingqi group, 16 km SE of Yakou, N28.72276°, E97.70598°, 3698 m, 1.IX.2014, Jincheng Liu leg. **Paratypes**: 3♂♂, 3♀♀ (IZCAS): same data as holotype; 3♂♂ (IZCAS): China: Tibet: Zayü: Zhowagoin Township, Xiongjiu Village, N28.60677°, E97.28166°, 1938 m, 29.VIII.2014, Jincheng Liu leg.

###### Etymology.

The specific name refers to the type locality, Sangjiu Village; adjective.

###### Diagnosis.

**Figure 1.**
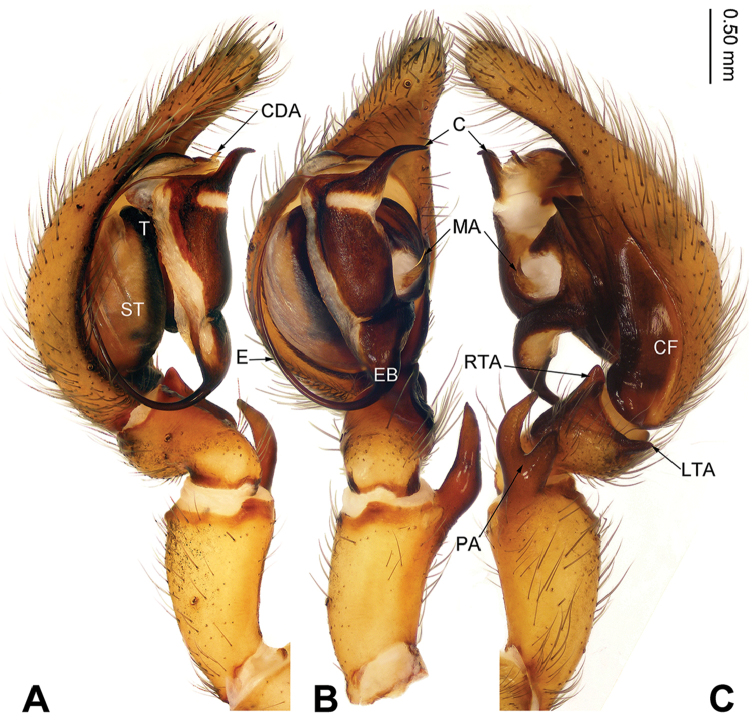
Left male palp of *Sinodraconarius sangjiuensis* sp. n., holotype. **A** Prolateral view **B** Ventral view **C** Retrolateral view. Scale bar: equal for **A, B, C**.

**Figure 2.**
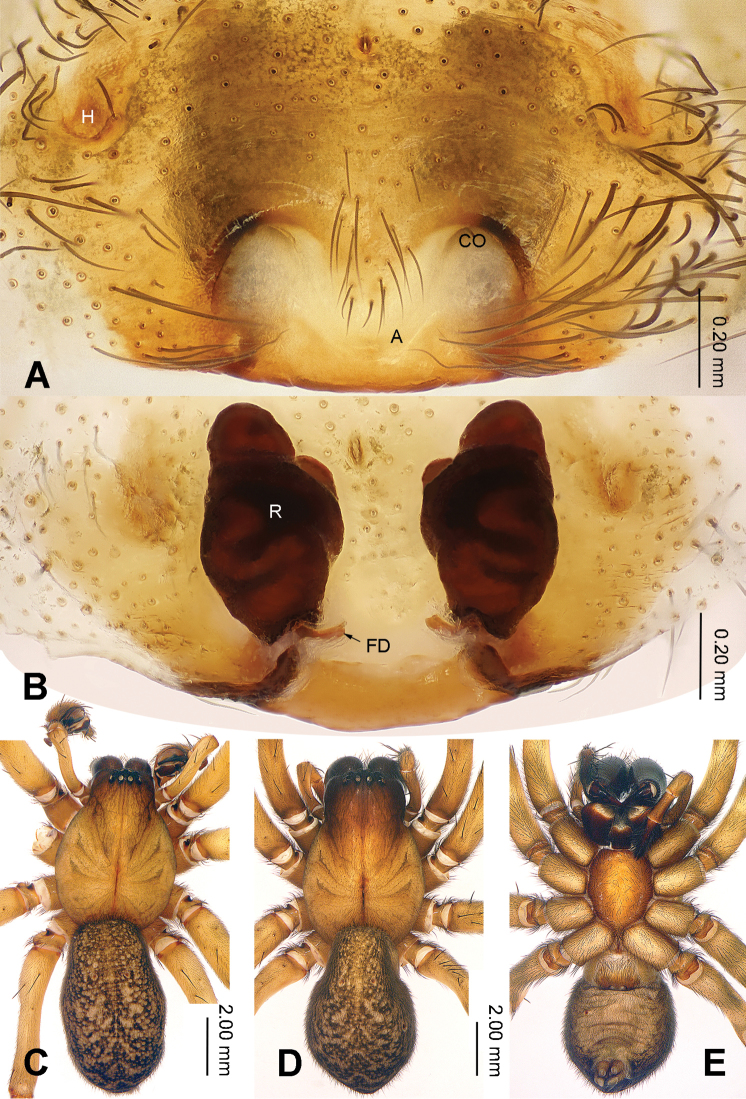
Epigyne and habitus of *Sinodraconarius sangjiuensis* sp. n. **A** Epigyne, ventral **B** Vulva, dorsal **C** Male habitus, dorsal **D** Female habitus, dorsal **E** Female habitus, ventral. Scale bar equal for **D** and **E**.

The males can be easily distinguished from other *Sinodraconarius* gen. n. species by the patellar apophysis longer than the tibia *vs.* shorter than the tibia in other species (Fig. 1A–C). The females can be easy distinguished from other *Sinodraconarius* gen. n. species by the epigynal hoods in the center of the epigynal plate *vs.* anterolaterally in other species (Fig. 2A–B).

###### Description.

**Male (holotype).** Total length 12.25. Carapace 5.75 long, 4.50 wide. Abdomen 6.50 long, 4.00 wide. Eye sizes and interdistances: AME 0.15, ALE 0.23, PME 0.20, PLE 0.23; AME–AME 0.10, AME–ALE 0.15, AME–PME 0.23, ALE–PLE 0, PME–PME 0.18, PME–PLE 0.20. Leg measurements: I 23.72 (7.69, 7.05, 5.77, 3.21); II 22.43 (7.69, 6.41, 5.45, 2.88); III 20.19 (6.73, 5.77, 5.13, 2.56); IV 24.67 (8.01, 7.05, 6.73, 2.88). Palp: patella longer than tibia; patellar apophysis thin and long, about three times longer than wide, with two branches and ventral branch larger than dorsal one; anterior 1/3 of RTA extending beyond the tibia, apex of RTA slightly bent; LTA about half of the RTA length; conductor short, apex of conductor pointed and bent retrolaterally; apex of median apophysis pointed; dorsal conductor apophysis broad, the visible part (between conductor and tegulum) subtriangular; embolus beginning at 5:30 o’clock position (Fig. 1A–C).

**Female (paratype).** Total length 12.50. Carapace 6.00 long, 4.25 wide. Abdomen 6.50 long, 4.25 wide. Eye sizes and interdistances: AME 0.15, ALE 0.25, PME 0.20, PLE 0.26; AME–AME 0.10, AME–ALE 0.10, AME–PME 0.25, ALE–PLE 0, PME–PME 0.15, PME–PLE 0.30. Leg measurements: I 18.59 (6.41, 6.09, 3.84, 2.25); II 18.27 (6.41, 5.77, 3.84, 2.25); III 17.45 (6.09, 5.27, 3.84, 2.25); IV 21.15 (6.41, 6.41, 5.45, 2.88). Epigyne: apex of the V-shaped septum tapering; atrium two times longer than wide, occupying approx. 1/8 of epigyne plate; copulatory ducts hidden by receptacles in ventral view, hidden by epigyne in dorsal view; receptacles broad and separated by 1/2 width of receptacle; head of receptacles located anteriorly, broad and short, 1/4 length and 1/6 width of receptacles (Fig. 2A–B).

###### Variation.

Total length of males 9.94–12.25 (n = 7) and of females 11.22–16.70 (n = 3).

###### Distribution.

Known only from Zayü, Tibet (Fig. 11).

##### Sinodraconarius cawarongensis

Taxon classificationAnimaliaAraneaeAgelenidae

Z. Zhao & S. Li sp. n.

http://zoobank.org/6A67E4D6-67A1-41BC-A22A-03D82C14E98F

Figs 3 , 4 , 11

###### Type material.

**Holotype** ♂ (IZCAS): China: Tibet: Zayü: Cawarong Township, 3.5 km E of Jumuchang, N28.55227°, E98.19554°, 3145 m, 7.IX.2014, Jincheng Liu leg. **Paratypes**: 13♂♂, 4♀♀ (IZCAS): same data as holotype; 9♂♂, 1♀ (IZCAS): China: Tibet: Zayü: Zhowagoin Township, Ridong Village, N28.49183°, E98.11320°, 3495 m, 4.IX.2014, Jincheng Liu; 6♂♂, 2♀♀ (IZCAS): China: Tibet: Zayü: Zhowagoin Township, 6 km N of Muruo Village, N28.59332°, E98.02774°, 3955 m, 5.IX.2014, Jincheng Liu leg.

###### Etymology.

The specific name refers to the type locality, Cawarong Township; adjective.

###### Diagnosis.

**Figure 3.**
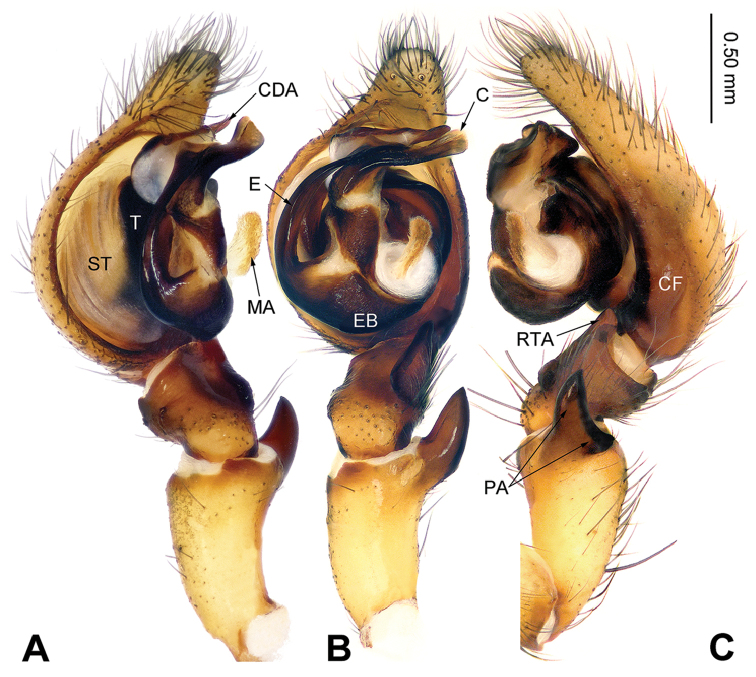
Left male palp of *Sinodraconarius cawarongensis* sp. n., holotype. **A** Prolateral view **B** Ventral view **C** Retrolateral view. Scale bar equal for **A, B, C**.

**Figure 4.**
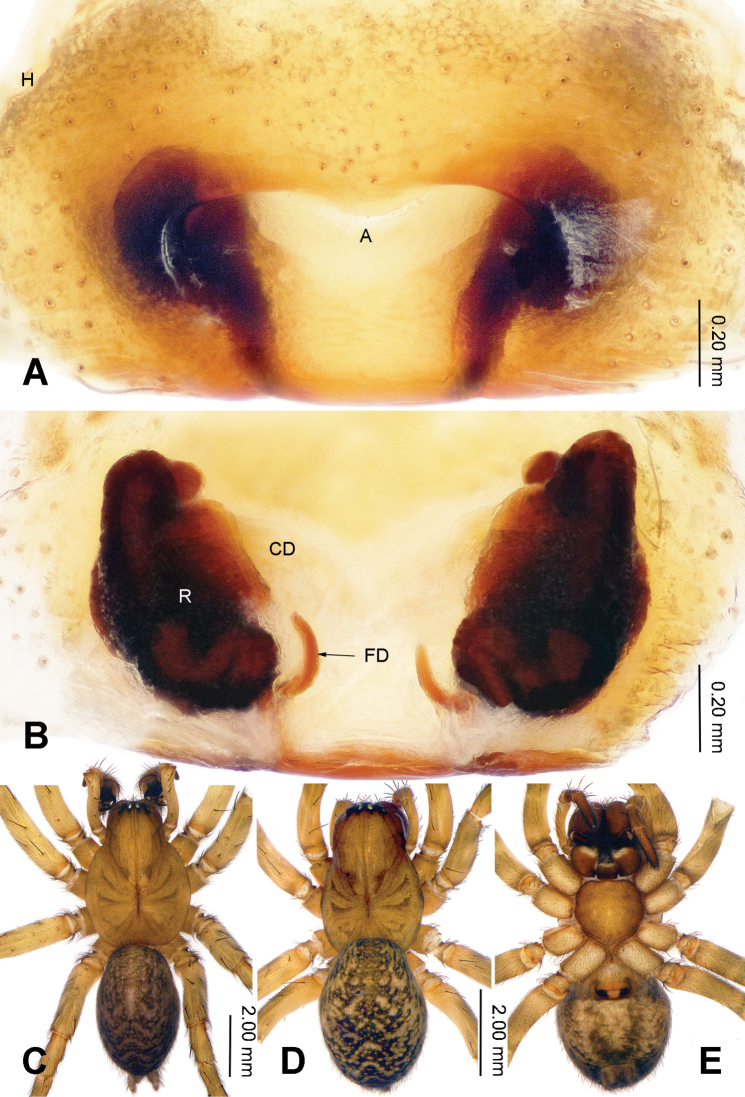
Epigyne and habitus of *Sinodraconarius cawarongensis* sp. n. **A** Epigyne, ventral **B** Vulva, dorsal **C** Male habitus, dorsal **D** Female habitus, dorsal **E** Female habitus, ventral. Scale bar equal for **D** and **E**.

The males are similar to *S. patellabifidus* by having an indistinct LTA and a long median apophysis but can be differentiated by the branches of the patellar apophysis, with the ventral branch larger than the dorsal branch in retrolateral view *vs.* the ventral branch equal to the dorsal branch in *S. patellabifidus* (Figs 3A–C, 7A–C). The females can be differentiated from *S. sangjiuensis* sp. n. by having the head of receptacles located anteriorly *vs.* mediolaterally in *S. sangjiuensis* sp. n. and the septum indistinct (apex of the septum is tapering in *S. sangjiuensis* sp. n.) (Figs 2A–B, 4A–B, 8A–B).

###### Description.

**Male (holotype).** Total length 8.45. Carapace 4.50 long, 3.35 wide. Abdomen 3.95 long, 2.65 wide. Eye sizes and interdistances: AME 0.12, ALE 0.18, PME 0.15, PLE 0.16; AME–AME 0.09, AME–ALE 0.05, AME–PME 0.15, ALE–PLE 0, PME–PME 0.13, PME–PLE 0.15. Leg measurements: I 14.86 (5.25, 4.81, 2.88, 1.92); II 13.99 (5.25, 4.25, 2.88, 1.61); III 13.73 (4.75, 3.85, 3.21, 1.92); IV 16.28 (5.45, 4.49, 4.10, 2.24). Palp: with one crescent-like bifurcate patellar apophysis, ventral branch is larger than dorsal branch of patellar apophysis; anterior 1/5 of RTA extending beyond the tibia; LTA indistinct; cymbial furrow less than 1/3 of cymbium length; apex of conductor pointed and bent retrolaterally; median apophysis finger-like, covered in short hairs; dorsal conductor apophysis broad, the visible part (between conductor and tegulum) subtriangular; embolus broad, beginning at position 8:30 o’clock (Fig. 3A–C).

**Female (paratype).** Total length 6.90. Carapace 3.40 long, 2.30 wide. Abdomen 3.50 long, 2.50 wide. Eye sizes and interdistances: AME 0.09, ALE 0.16, PME 0.14, PLE 0.15; AME–AME 0.08, AME–ALE 0.05, AME–PME 0.18, ALE–PLE 0, PME–PME 0.13, PME–PLE 0.14. Leg measurements: I 8.68 (3.30, 2.75, 1.66, 0.97); II 8.16 (3.05, 2.49, 1.66, 0.96); III 7.98 (3.15, 2.24, 1.61, 0.98); IV 9.82 (3.75, 2.75, 2.11, 1.21). Epigyne: rectangular; septum indistinct; hoods located anterolaterally on the plate; atrium 3 times longer than wide, anterior part slightly wider than posterior part, occupying about 1/8 of epigynal plate; receptacles broad, separated by a width of a receptacle; the head of receptacles located anteriorly (Fig. 4A–B).

###### Variation.

Total length of males (n = 29) 7.69–10.26 and of females (n = 7) 6.90–8.34.

###### Distribution.

Known only from Zayü, Tibet (Fig. 11).

##### Sinodraconarius muruoensis

Taxon classificationAnimaliaAraneaeAgelenidae

Z. Zhao & S. Li sp. n.

http://zoobank.org/DBBFF4FB-ECD3-40E6-8BAC-663C0342FC89

Figs 5 , 6 , 11

###### Type material.

**Holotype** ♂ (IZCAS): China: Tibet: Zayü: Zhowagoin Township, Muruo Village, Gaoshan Mountain pasture, N28.62049°, E98.05035°, 4347 m, 5.IX.2014, Jincheng Liu leg. **Paratypes**: 5♂♂, 3♀♀ (IZCAS): same data as holotype; 1♂, 5♀♀ (IZCAS): China: Tibet: Zayü: Zhowagoin Township, Muruo Village, Qimala Yakou, N28.62049°, E98.05035°, 4657 m, 2.IX.2014, Jincheng Liu leg.

###### Etymology.

The specific name refers to the type locality, Muruo Village; adjective.

###### Diagnosis.

**Figure 5.**
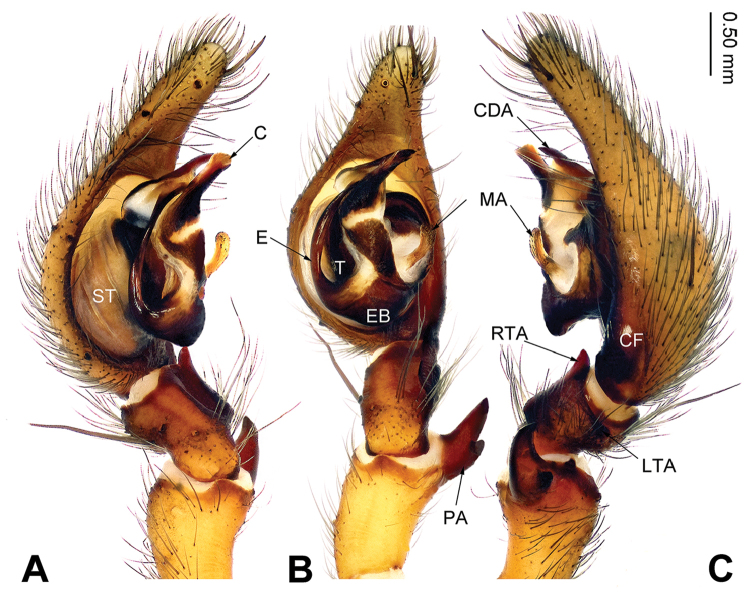
Left male palp of *Sinodraconarius muruoensis* sp. n., holotype. **A** Prolateral view **B** Ventral view **C** Retrolateral view. Scale bar equal for **A, B, C**.

**Figure 6.**
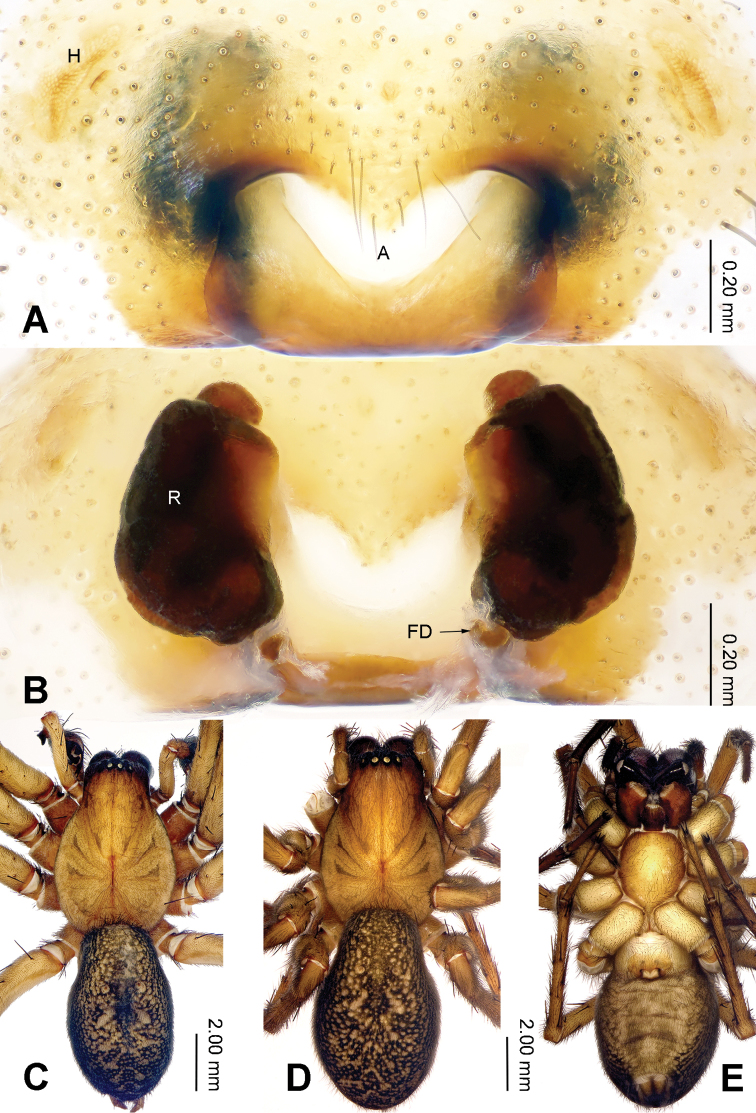
Epigyne and habitus of *Sinodraconarius muruoensis* sp. n. **A** Epigyne, ventral **B** Vulva, dorsal **C** Male habitus, dorsal **D** Female habitus, dorsal **E** Female habitus, ventral. Scale bar equal for **D** and **E**.

The males of the new species are similar to these of *S. sangjiuensis* sp. n. by having a ventral branch of the patellar apophysis that is larger than the dorsal branch but can be easily distinguished from *S. sangjiuensis* sp. n. by the apex of conductor being straight *vs.* bent in *S. sangjiuensis* sp. n. (Figs 1A–C, 5A–C). The females of the new species are similar to *S. sangjiuensis* sp. n. by the apex of septum tapering but can be easily distinguished from by having the hoods and the head of the receptacles located anteriorly rather than medially (Figs 2A–B, 6A–B).

###### Description.

**Male (holotype).** Total length 13.50. Carapace 6.50 long, 4.50 wide. Abdomen 7.00 long, 4.00 wide. Eye sizes and interdistances: AME 0.10, ALE 0.23, PME 0.20, PLE 0.25; AME–AME 0.13, AME–ALE 0.15, AME–PME 0.30, ALE–PLE 0, PME–PME 0.20, PME–PLE 0.15. Leg measurements: I 27.84 (8.64, 8.63, 6.73, 3.84); II 27.21 (8.63, 8.65, 6.41, 3.52); III 24.99 (8.01, 7.05, 6.41, 3.52); IV 29.48 (9.62, 8.02, 8.01, 3.83). Palp: with one bifurcate patellar apophysis (ventral branch of patellar apophysis larger than dorsal one) and one small apophysis; LTA indistinct; cymbial furrow short, about 1/5 of the of cymbial length; conductor short, extending anteriorly, the apex of conductor pointed and bending retro-anteriorly; median finger-like; dorsal the visible part of conductor apophysis (between conductor and tegulum) subtriangular, shorter than conductor; embolus beginning at position 7:30 o’clock (Fig. 5A–C).

**Female (paratype).** Total length 11.00. Carapace 5.00 long, 4.00 wide. Abdomen 6.00 long, 4.00 wide. Eye sizes and interdistances: AME 0.15, ALE 0.22, PME 0.20, PLE 0.24; AME–AME 0.10, AME–ALE 0.10, AME–PME 0.15, ALE–PLE 0, PME–PME 0.15, PME–PLE 0.25. Leg measurements: I 16.88 (6.08, 5.44, 3.12, 2.24); II 15.69 (6.08, 4.48, 3.21, 1.92); III 15.40 (5.77, 4.49, 3.23, 1.91); IV 18.58 (6.73, 5.12, 4.81, 1.92). Epigyne: with V-shaped septum, apex of septum tapering; hoods located laterally; atrium two times wider than long, occupying approx. 1/7 of epigynal plate; receptacles separated by the width of a receptacle; the head of the receptacles broad, short, located anteriorly (Fig. 6A–B).

###### Variation.

Total length of males (n = 7) 11.86–17.60 and of females (n = 8) 6.90–11.00.

###### Distribution.

Known only from Zayü, Tibet (Fig. 11).

##### Sinodraconarius patellabifidus

Taxon classificationAnimaliaAraneaeAgelenidae

(Wang, 2003) comb. n.

Figs 7 , 8 , 11

Draconarius patellabifidus Wang 2003: 542, fig. 49A–D (♂♀); Wang et al. 2010: 81, figs 331–351 (♂♀); Zhu et al. 2017: 338, fig. 209A–D (♂♀).

###### Material examined.

2♀♀ (IZCAS): China: Yunnan Province: Nujiang Lisu Autonomous Prefecture: Lushui County, Liuku Township, N25.80797°, E98.84226°, 1220 m,18.IX.2014, Jincheng Liu leg.; 1♂, 1♀ (IZCAS): China: Yunnan Province: Nujiang Lisu Autonomous Prefecture: Lushui County, Luzhang Township, Fengxue Yakou, N25.97244°, E98.68376°, 3150 m, 19.IX.2014, Jincheng Liu leg.

###### Diagnosis.

**Figure 7.**
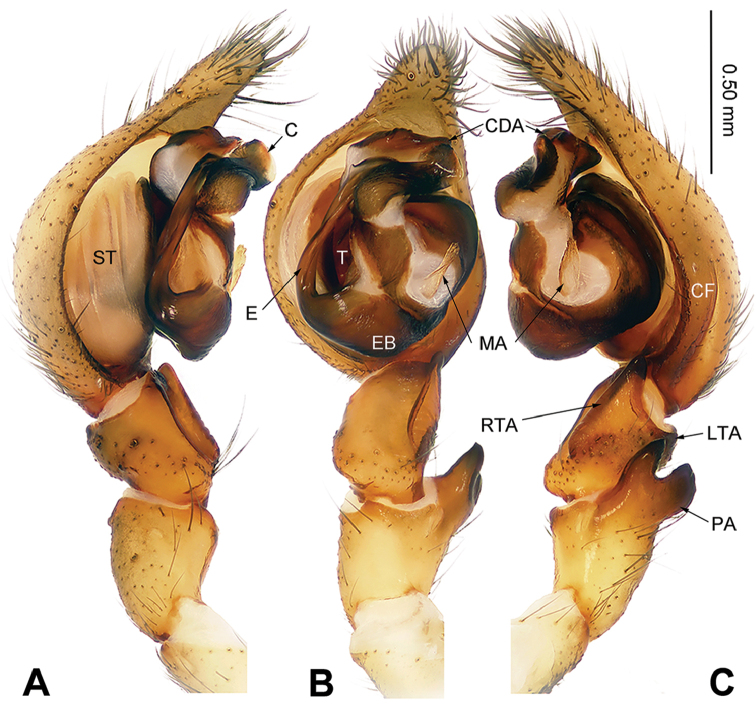
Left male palp of *Sinodraconarius patellabifidus* **A** Prolateral view **B** Ventral view **C** Retrolateral view. Scale bar equal for **A, B, C**.

**Figure 8.**
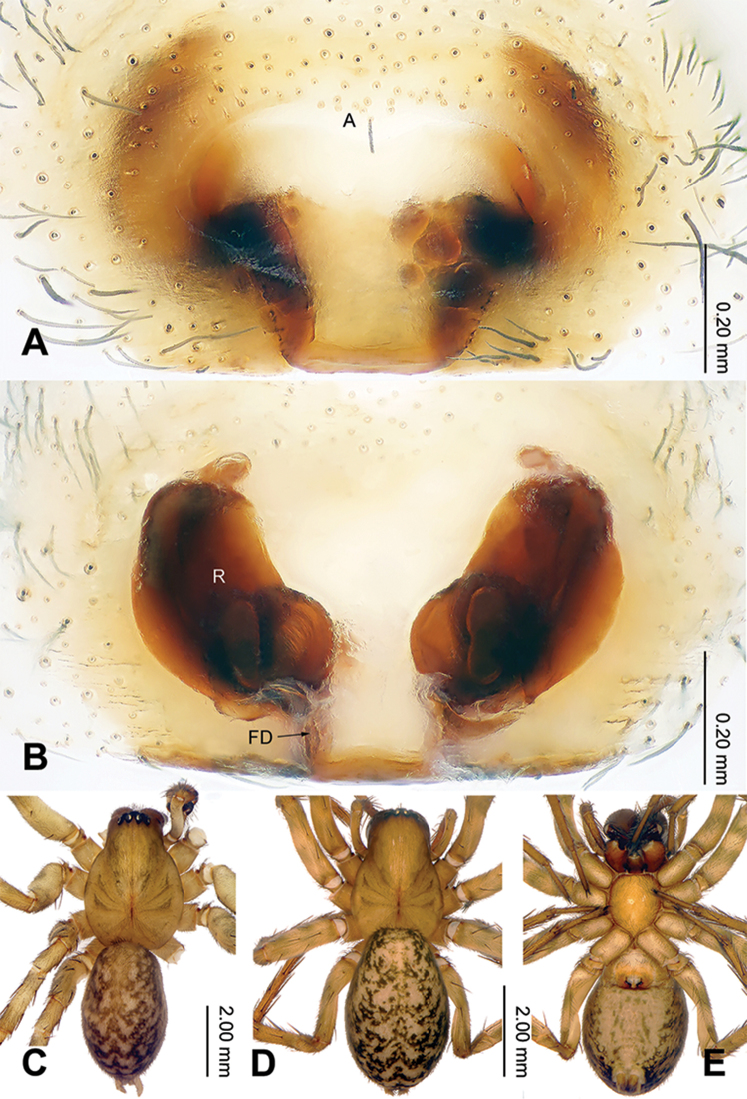
Epigyne and habitus of *Sinodraconarius patellabifidus* **A** Epigyne, ventral **B** Vulva, dorsal **C** Male habitus, dorsal **D** Female habitus, dorsal **E** Female habitus, ventral. Scale bar equal for **D** and **E**.

The males can be differentiated from *S. sangjiuensis* sp. n. by the ventral branch of the patellar apophysis which is the same length as the dorsal branch *vs.* larger than dorsal one in *S. sangjiuensis* sp. n. (Figs 1A–C, 7A–C). The females can be differentiated from *S. sangjiuensis* sp. n. by the head of the receptacles located anteriorly *vs.* mediolaterally in *S. sangjiuensis* sp. n.; septum indistinct (apex of the septum is tapering in *S. sangjiuensis* sp. n.) (Figs 2A–B, 8A–B).

###### Description.

Described by Wang (2003).

###### Variation.

Total length of females (n = 3) 8.75–11.80.

###### Distribution.

Known only from Yunnan (Fig. 11; Wang 2003: map 17; Wang et al. 2010: 545).

##### Sinodraconarius yui

Taxon classificationAnimaliaAraneaeAgelenidae

Z. Zhao & S. Li sp. n.

http://zoobank.org/1BB98FE5-7F5A-4CA3-8DDF-1D1C79E52CD8

Figs 9 , 10 , 11

Coelotes himalayaensis Hu 2001: 134, fig. 45.3–4 (♂ only, ♀ mismatched).
Draconarius himalayaensis Wang 2003: 534 (♂ only, ♀ mismatched); Zhu et al. 2017: 289, fig. 166C–D (♂ only, ♀ mismatched)

###### Type material.

**Holotype** ♂ (IZCAS): China: Tibet: Nyingchi: Segula Mountain, N29.605017°, E94.609117°, 4184 ± 4 m, 11.X.2010, Hao Yu leg. **Paratypes**: 2♂♂ (IZCAS): same data as holotype; 1♀ (IZCAS): China: Tibet: Nyingchi: Positive face of Segula Mountain, N29.607583°, E94.608767°, 4190 ± 13 m, 12.VIII.2010, Hao Yu leg.

###### Etymology.

The specific name is after Hao Yu, the collector of specimens used in this study; noun (name) in genitive case.

###### Diagnosis.

**Figure 9.**
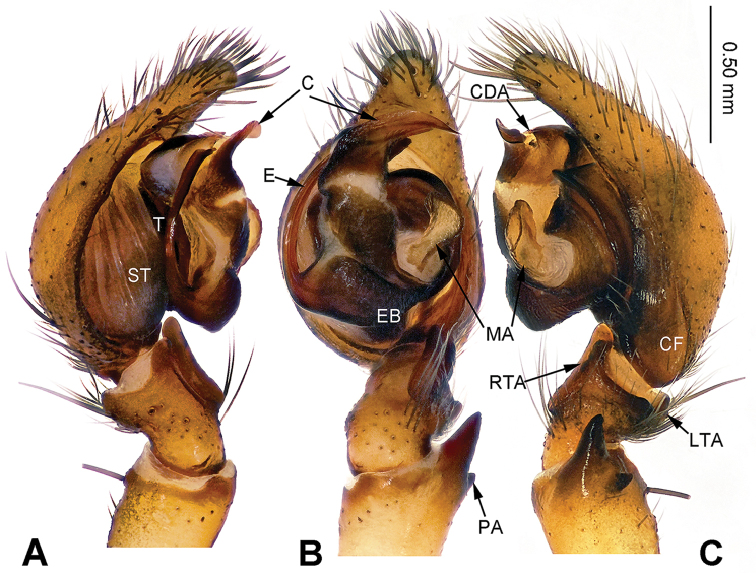
Left male palp of *Sinodraconarius yui* sp. n., holotype. **A** Prolateral view **B** Ventral view **C** Retrolateral view. Scale bar equal for **A, B, C**.

**Figure 10.**
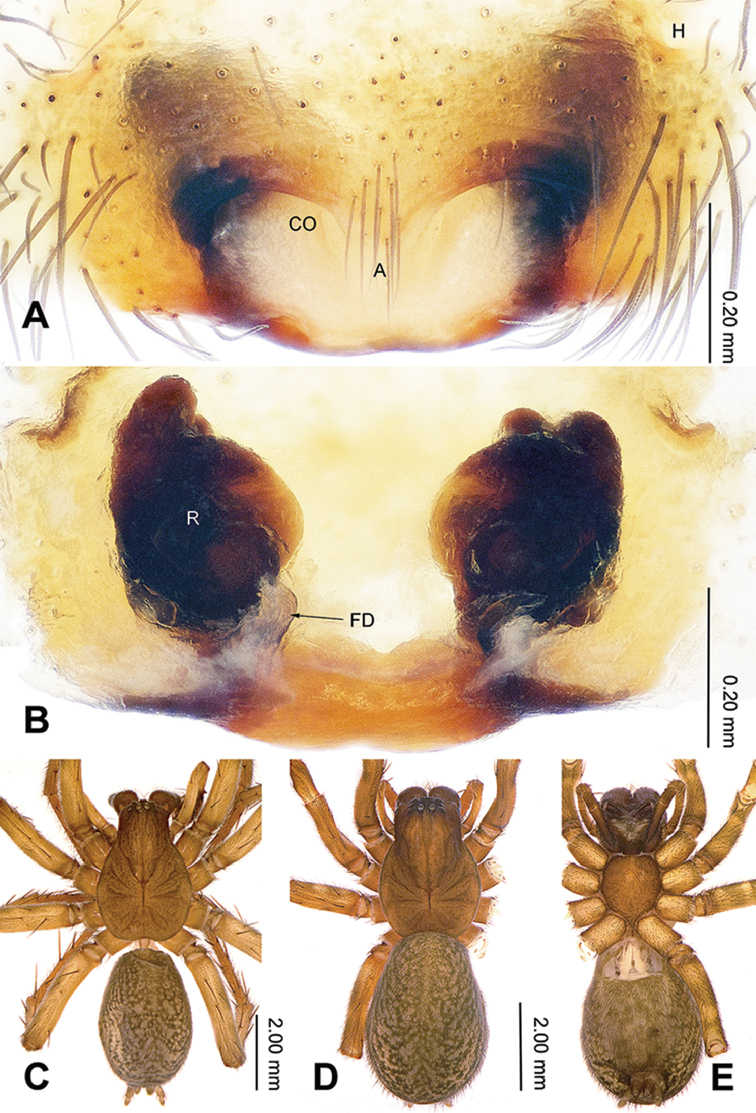
Epigyne and habitus of *Sinodraconarius yui* sp. n. **A** Epigyne, ventral **B** Vulva, dorsal **C** Male habitus, dorsal **D** Female habitus, dorsal **E** Female habitus, ventral. Scale bar equal for **D** and **E**.

The males are similar to these of *S. sangjiuensis* sp. n. by the ventral branch of the patellar apophysis larger than dorsal one and the apex of the conductor pointed and bent retrolaterally, but can be easily distinguished from *S. sangjiuensis* sp. n. by the short palp (Figs 1A–C, 9A–C). The females are similar to *S. sangjiuensis* sp. n. by having the apex of the septum tapering but can be easily distinguished from *S. sangjiuensis* sp. n. by the epigynal hoods located anterolaterally *vs.* mediolaterally in *S. sangjiuensis* sp. n. (Figs 2A–B, 10A–B).

###### Description.

**Male (holotype).** Total length 7.59. Carapace 4.00 long, 2.60 wide. Abdomen 3.59 long, 2.56 wide. Eye sizes and interdistances: AME 0.09, ALE 0.20, PME 0.14, PLE 0.19; AME–AME 0.08, AME–ALE 0.05, AME–PME 0.16, ALE–PLE 0, PME–PME 0.09, PME–PLE 0.13. Leg measurements: I 12.15 (4.06, 4.00, 2.68, 1.41); II 10.22 (3.44, 3.50, 2.03, 1.25); III 9.99 (3.28, 3.00, 2.40, 1.31); IV 12.82 (4.06, 3.80, 3.40, 1.56). Palp: ventral branch of patellar apophysis larger than dorsal one; anterior 1/5 part of RTA extending beyond the tibia; LTA obvious; cymbial furrow less than 1/4 of cymbium length; the apex of conductor concave and spiral; median apophysis conspicuous, finger-like, the apex of the median apophysis expanded and open; dorsal conductor apophysis broad, the visible part (between conductor and tegulum) hidden by conductor in ventral view; embolus beginning at position 10 o’clock (Fig. 9A–C).

**Female (paratype).** Total length 7.89. Carapace 3.40 long, 2.40 wide. Abdomen 4.49 long, 2.40 wide. Eye sizes and interdistances: AME 0.10, ALE 0.18, PME 0.15, PLE 0.15; AME–AME 0.11, AME–ALE 0.06, AME–PME 0.15, ALE–PLE 0, PME–PME 0.10, PME–PLE 0.15. Leg measurements: I 9.17 (3.58, 2.88, 1.75, 0.96); II 8.66 (3.07, 2.88, 1.75, 0.96); III 8.47 (3.20, 2.56, 1.75, 0.96); IV 11.05 (3.59, 3.52, 2.50, 1.44). Epigyne: apex of the V-shaped septum tapering; hoods located laterally; atrium two times wider than long, occupying approx. 1/4 of epigynal plate; receptacles spaced by the width of a receptacle; the head of the receptacles broad and short, located anteriorly (Fig. 10A–B).

###### Comments.

The male of the new species was first described by Hu (2001) as the allotype of *Coelotes himalayaensis* and later transferred from *Coelotes* to *Draconarius* by Wang (2003). However, we found that the male and female of *D. himalayaensis* were mismatched. Therefore, the male is established as a new species here, and both sexes are described.

###### Variation.

Total length of males (n = 3) 7.05–7.59.

###### Distribution.

**Figure 11.**
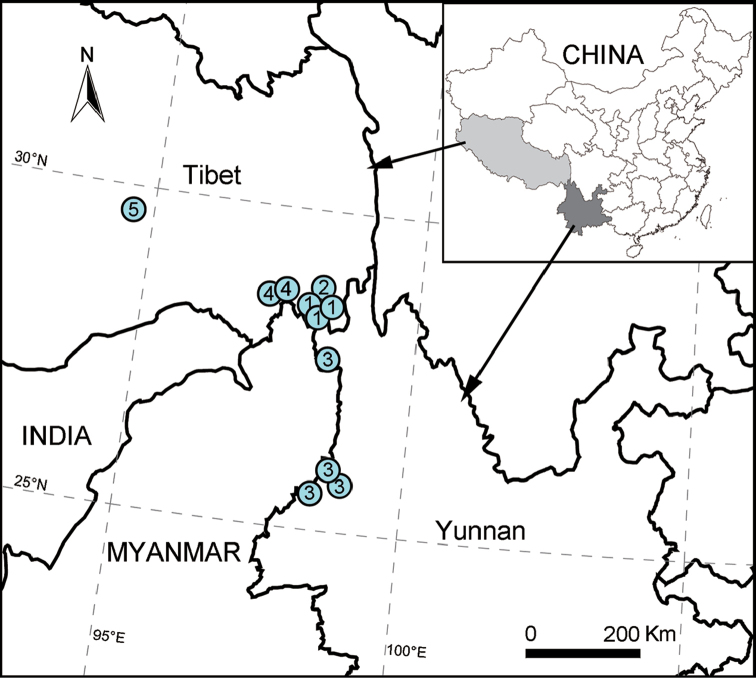
Localities of *Sinodraconarius* species in the Southwest China. **1** *S. cawarongensis* sp. n. **2** *S. muruoensis* sp. n. **3** *S. patellabifidus* **4** *S. sangjiuensis* sp. n. **5** *S. yui* sp. n.

Known only from Nyingchi, Tibet (Fig. 11).

## Supplementary Material

XML Treatment for Sinodraconarius

XML Treatment for Sinodraconarius sangjiuensis

XML Treatment for Sinodraconarius cawarongensis

XML Treatment for Sinodraconarius muruoensis

XML Treatment for Sinodraconarius patellabifidus

XML Treatment for Sinodraconarius yui

## References

[B1] Chen L Li S Zhao Z (2015) A new genus of Coelotinae (Araneae, Agelenidae) from southern China. ZooKeys 541: 41–56. 10.3897/zookeys.541.6678 PMC4714376 26798279

[B2] Chen L Zhao Z Li S (2016) *Sinocoelotes* gen. n., a new genus of the subfamily Coelotinae (Araneae, Agelenidae) from Southeast Asia. ZooKeys 614: 51–86. 10.3897/zookeys.614.8663 PMC5027656 27667932

[B3] Hu JL (2001) Spiders in Qinghai-Tibet Plateau of China. Henan Science and Technology Publishing House, 658 pp.

[B4] Koch CL (1837) Übersicht des Arachnidensystems. Nürnberg, Heft 1, 1–39. 10.5962/bhl.title.39561

[B5] Ovtchinnikov SV (1999) On the supraspecific systematics of the subfamily Coelotinae (Araneae, Amaurobiidae) in the former USSR fauna. Tethys Entomological Research 1: 63–80.

[B6] Pickard-Cambridge FO (1893) Handbook to the study of British spiders (Drassidae and Agalenidae). British Naturalist 3: 117–170.

[B7] Wang XP (2002) A generic-level revision of the spider subfamily Coelotinae (Araneae, Amaurobiidae). Bulletin of the American Museum of Natural History 269: 1–150. 10.1206/0003-0090(2002)269<0001:AGLROT>2.0.CO;2

[B8] Wang XP (2003) Species revision of the coelotine spider genera *Bifidocoelotes*, *Coronilla*, *Draconarius*, *Femoracoelotes*, *Leptocoelotes*, *Longicoelotes*, *Platocoelotes*, *Spiricoelotes*, *Tegecoelotes*, and *Tonsilla* (Araneae: Amaurobiidae). Proceedings of the California Academy of Sciences 54: 499–662.

[B9] Wang XP Griswold CE Miller JA (2010) Revision of the genus *Draconarius* Ovtchinnikov 1999 (Agelenidae: Coelotinae) in Yunnan, China, with an analysis of the Coelotinae diversity in the Gaoligongshan Mountains. Zootaxa 2593: 1–127. 10.11646/zootaxa.2593.1.1

[B10] World Spider Catalog (2018) Natural History Museum Bern. Version 18.5. http://wsc.nmbe.ch [accessed 4 January 2018]

[B11] Zhao Z Li S (2016) *Papiliocoelotes* gen. n., a new genus of Coelotinae (Araneae, Agelenidae) spiders from the Wuling Mountains, China. ZooKeys 585: 33–50. 10.3897/zookeys.585.8007 PMC4857037 27199603

[B12] Zhao Z Li S (2017) Extinction vs. rapid radiation: the juxtaposed evolutionary histories of coelotine spiders support the Eocene-Oligocene orogenesis of the Tibetan Plateau. Systematic Biology 66(6): 988–1006. 10.1093/sysbio/syx042 28431105

[B13] Zhu MS Wang XP Zhang ZS (2017) Fauna Sinica: Invertebrata Vol. 59: Arachnida: Araneae: Agelenidae and Amaurobiidae. Science Press, Beijing, 727 pp.

